# Associations of COVID-19-related fear with kidney disease quality of life and its subscales among hemodialysis patients as modified by health literacy: a multi-hospital survey

**DOI:** 10.1080/21642850.2024.2376585

**Published:** 2024-07-12

**Authors:** Minh D. Pham, Tu T. Tran, Tuyen Van Duong, Binh N. Do, Loan T. Dang, Dung H. Nguyen, Trung A. Hoang, Hoang C. Nguyen, Lan T. H. Le, Linh V. Pham, Lien T. H. Nguyen, Hoi T. Nguyen, Nga T. Trieu, Thinh V. Do, Manh V. Trinh, Tung H. Ha, Dung T. Phan, Thao T. P. Nguyen, Kien T. Nguyen

**Affiliations:** a Department of Nutrition, Military Hospital 103, Hanoi, Vietnam; b Department of Nutrition, Vietnam Military Medical University, Hanoi, Vietnam; c International Ph.D. Program in Medicine, College of Medicine, Taipei Medical University, Taipei, Taiwan; d Department of Internal Medicine, Thai Nguyen University of Medicine and Pharmacy, Thai Nguyen, Vietnam; e Department of Nephro-Urology and Dialysis, Thai Nguyen National Hospital, Thai Nguyen City, Vietnam; f School of Nutrition and Health Sciences, Taipei Medical University, Taipei, Taiwan; g Department of Military Science, Vietnam Military Medical University, Hanoi, Vietnam; h Department of Infectious Diseases, Vietnam Military Medical University, Hanoi, Vietnam; i Faculty of Nursing and Midwifery, Hanoi Medical University, Hanoi, Vietnam; j School of Nursing, National Taipei University of Nursing and Health Sciences, Taipei, Taiwan; k Thoracic and Vascular surgery department, Bach Mai Hospital, Hanoi, Vietnam; l Hemodialysis Department, Nephro-Urology-Dialysis Center, Bach Mai Hospital, Hanoi, Vietnam; m Director Office, Thai Nguyen National Hospital, Thai Nguyen City, Vietnam; n President Office, Thai Nguyen University of Medicine and Pharmacy, Thai Nguyen City, Vietnam; o Training and Direction of Healthcare Activity Center, Thai Nguyen National Hospital, Thai Nguyen City, Vietnam; p Biochemistry Department, Thai Nguyen National Hospital, Thai Nguyen City, Vietnam; q Department of Pulmonary & Cardiovascular Diseases, Hai Phong University of Medicine and Pharmacy Hospital, Hai Phong, Vietnam; r President Office, Hai Phong University of Medicine and Pharmacy, Hai Phong, Vietnam; s Director Office, Hai Phong International Hospital, Hai Phong, Vietnam; t Hemodialysis Division, Hai Phong International Hospital, Hai Phong, Vietnam; u Director Office, Bai Chay Hospital, Ha Long, Vietnam; v Director Office, Quang Ninh General Hospital, Ha Long, Vietnam; w Director Office, General Hospital of Agricultural, Hanoi, Vietnam; x Faculty of Nursing, Hanoi University of Business and Technology, Hanoi, Vietnam; y Nursing Office, Thien An Obstetrics and Gynecology Hospital, Hanoi, Vietnam; z Institute for Community Health Research, University of Medicine and Pharmacy, Hue University, Hue, Vietnam; aa Department of Health Promotion, Faculty of Social and Behavioral Sciences, Hanoi University of Public Health, Hanoi, Vietnam

**Keywords:** Fear of COVID-19, health literacy, mental component summary, physical component summary, kidney disease component summary

## Abstract

**Background:**

Receiving hemodialysis treatment makes end-stage renal disease (ESRD) patients highly vulnerable amidst the COVID-19 pandemic. Hence, their kidney disease quality of life (KDQOL) is affected. We aimed to examine the association between fear of COVID-19 (FCoV-19) and KDQOL, and the effect modification of Health literacy (HL) on this association.

**Material and Methods:**

A survey was conducted at 8 hospitals from July 2020 to March 2021 on 972 patients. Data collection includes socio-demographic factors, clinical parameters, HL, digital healthy diet literacy (DDL), hemodialysis diet knowledge (HDK), FCoV-19, suspected COVID-19 symptoms (S-COVID-19-S), and KDQOL.

**Results:**

Higher HL scores B = 0.13 (95% CI = 0.06–0.21, *p* = 0.001) and HDK scores B = 0.58 (95% CI = 0.31–0.85, *p* = 0.001) were associated with higher KDQOL scores. Whereas, S-COVID-19-S B = −6.12 (95% CI = −7.66 to – 4.58, *p* = 0.001) and FCoV-19 B = −0.91 (95% CI = −1.03 to – 0.80, *p* = 0.001) were associated with lower KDQOL scores. Notably, higher HL scores significantly attenuate the negative impact of FCoV-19 on overall KDQOL and the kidney disease component summary.

**Conclusions:**

In hemodialysis patients, FCoV-19 and S-COVID-19-S were associated with a lower KDQOL. Health literacy significantly mitigates the negative impact of FCoV-19 on KDQOL. Strategic public health interventions to improve HL are suggested to protect patient’s KDQOL during the pandemic.

## Introduction

The COVID-19 pandemic heavily affected individuals’ physical and mental well-being worldwide (Chen et al., 2022; WHO, 2022). Within vulnerable populations such as patients undergoing hemodialysis, the fear and anxiety stemming from COVID-19 can impair their quality of life (QOL) (Nguyen et al., 2021; Park & Park, 2023), and exacerbate their existing health concerns (Fishbane et al., 2015). Understanding the factors that modify the impact of fear on the quality of life in hemodialysis patients is crucial to developing targeted interventions and support strategies.

Health literacy is a concept that encompasses an individual's knowledge, motivation, and skills to access, understand, evaluate, and use health information effectively (Sørensen et al., 2012). It has been shown to influence patients’ ability to navigate the healthcare system, make informed decisions, and effectively manage their health conditions (Al Sayah et al., 2013). Given the complexity of hemodialysis treatment and the need for self-care management, HL was particularly important for hemodialysis patients in the context of fear related to COVID-19.

While previous studies examined the impact of COVID-19-related fear on the quality of life in various populations, limited research focused on hemodialysis patients (Beisland et al., 2021; Nguyen et al., 2021; Yeni et al., 2022). Furthermore, the potential moderating role of HL remains to be explored. Therefore, we aimed to investigate the negative impact of COVID-19-related fear on kidney disease quality of life and its subscales, and explore the potential modifying effect of HL on that association in hemodialysis patients.

## Methods

### Study design and participants

From July 2020 to March 2021, a cross-sectional study was conducted at eight hospitals in Vietnam, including four at the national level and four at the provincial level, to ensure representation from various regions in Vietnam. Eligible participants were hemodialysis patients aged between 18 and 85 who could read and respond to the survey. Patients who had received hemodialysis treatment for less than 3 months were excluded from the study. Importantly, no positive cases of COVID-19 were reported among the patients during the study period. Prior to participating in the research, patients were required to provide informed consent. This study was approved by the Institutional Review Board of Hanoi University of Public Health in Vietnam (IRB No. 225/2020/YTCC-HD3). The study included a total sample size of 972 patients.

Data collection was described in previous studies (Dang et al., 2022; Le et al., 2023). In brief, a team comprising nephrologists, nurses, and students underwent training to ensure consistent and accurate data collection. Strict COVID-19 control measures, such as the mandatory use of masks, regular hand washing, and maintenance of physical distancing, were implemented throughout the study. The survey took approximately 30 minutes to be completed. Next, the collected data underwent a rigorous process of coding, cleaning, and analysis. This involved assigning appropriate codes to the data, identifying and rectifying any inconsistencies or errors, and preparing the dataset for analysis.

### Measurements

#### Outcome variables

The KDQOL was evaluated using the KDQOL 36-item short-form survey (KDQOL-36; version 1.3), developed by RAND Corporation (Health, 2012), which was validated in Vietnam (Vo et al., 2018; Vu et al., 2021). The KDQOL-36 consists of 5 distinct subscales: the physical component summary (PCS), mental component summary (MCS), burden of kidney disease (BKD), symptoms and problems of kidney disease (SPKD), and effects of kidney disease (EKD). The kidney disease component summary (KDCS) consists of BKD, SPKD, and EKD. Therefore, the overall KDQOL and three scales (MCS, PCS, and KDCS) were analyzed in our study, with the score potentially ranging from 0 to 100 (Cohen et al., 2019; Manavalan et al., 2017), with higher scores indicating better KDQOL (Ishiwatari et al., 2020; Jung et al., 2019).

#### Fear of COVID-19

The fear of COVID-19 was assessed using the 7-item Fear of COVID-19 Scale (FCoV-19S). Respondents were asked to indicate their level of agreement with each item on a scale ranging from 1 to 5, where 1 corresponds to ‘strongly disagree,’ 2 to ‘disagree,’ 3 to ‘neither agree nor disagree,’ 4 to ‘agree,’ and 5 to ‘strongly agree.’ The composite score encompasses values between 7 and 35, with higher scores denoting a greater sense of fear (Nguyen, Do et al., 2020). This tool was validated in Vietnam (Doan et al., 2021; Nguyen et al., 2022).

#### Health literacy and digital healthy diet literacy

The HL and DDL indices were evaluated using the 12-item short form of the Health Literacy Questionnaire (HLS-SF12) (Duong et al., 2019) and the Digital Healthy Diet Literacy Scale (DDL-4) (Duong et al., 2020), respectively. Respondents rated the perceived difficulty of each item on a 4-point scale, with 1 representing ‘very difficult,’ 2 for ‘fairly difficult,’ 3 for ‘fairly easy,’ and 4 for ‘very easy.’ The HL and DDL indices were derived using the following formula:

Index=(Mean−1)×(50/3)

In this equation, ‘Index’ stands for the specific index calculated, ‘Mean’ denotes the mean of all item ratings for each participant. The scores range from 0 to 50, with higher scores indicating elevated levels of HL or DDL. These scales have been validated and used in Vietnam (Sørensen et al., 2015; Van Hoa et al., 2020).

#### Covariates

Patient demographic information, such as age, gender, education, working status, marital status, and social status, along with medication payment ability, were collected. Social status was determined through a self-reported question asking participants to assess their social status based on factors such as education, occupation, and income, with response options including ‘low,’ ‘middle,’ and ‘high.’

Clinical parameters were assessed, including comorbidity, suspected COVID-19 symptoms (S-COVID-19-S), hemodialysis vintage (HD vintage, length of time on dialysis) (Chertow et al., 2000), edema, hypothyroidism, hyperthyroidism, hospitalization with one month and fear of COVID-19. S-COVID-19-S was identified if participants exhibited any of the following symptoms: fever, cough, fatigue, dyspnea, myalgia, sputum production/expectoration, sore throat, runny nose, confusion, headache, chest pain, rhinorrhea, diarrhea, and/or nausea/vomiting (Practice, 2020). Body mass index (BMI) was classified into 2 levels using the cutoff value at 24 kg/m^2^. This cutoff value was also used in previous studies on hemodialysis patients (Dang et al., 2022; Le et al., 2023). Physical activiy was assessed using the International Physical Activity Questionnaire short version (IPAQ-SF). The details were described in our previous publications (Dang et al., 2022; Le et al., 2023). The physical activity score (metabolic equivalent task-MET) was calculated by multiplying minutes spent on activities (sitting, walking, moderate, and vigorous activity) by 1.0, 3.3, 4.0, and 8.0, respectively. The MET was scored in minutes per week (MET-min/wk) and classified by tertiles.

Comorbidities were assessed using the Charlson comorbidity index (CCI) items which were modified in ESRD patients (Hemmelgarn et al., 2003). Each comorbid condition was assigned a specific score, e.g. 1 point was given to Peripheral vascular disease, Dementia, Chronic lung disease, Rheumatologic disease, Peptic ulcer disease, Diabetes with complications; 2 points were given to Myocardial infarction, Congestive heart failure, Cerebral vascular disease, Diabetes, Moderate/severe liver disease, Leukemia; 5 points were given to Lymphoma; and 10 points were given to Metastatic disease. The total score is calculated by summing up the scores of all the comorbidities present in a patient. The higher the score, the higher the predicted risk of mortality (Charlson et al., 2022).

Hemodialysis dietary knowledge (HDK) was evaluated using a 10-question scale that assessed participants’ knowledge about the hemodialysis diet, specifically focusing on water, potassium, phosphorus, sodium, and protein (Ryu et al., 2014). Each question had three response options: ‘correct,’ ‘incorrect,’ and ‘not sure.’ The correct answer was treated as ‘correct’, 1 point was given, and incorrect or ‘not sure’ answers were treated as ‘incorrect’, 0 point was given. The average score ranged from 0 to 1, with higher scores indicating greater knowledge. This questionnaire has been validated and utilized in previous research in hemodialysis patients (Le et al., 2022).

### Statistical analysis

The distributions of variables were analyzed and presented as count (n), percentage (%), mean, and standard deviation (SD) appropriately. Group differences in KDQOL scores were explored using t-tests or one-way ANOVA tests. To identify factors associated with KDQOL and its subscales, bivariate linear regression analysis was conducted. Based on a simulation study on confounder-selection strategies, factors showing an association with KDQOL at a *p*-value < 0.2 in the bivariate model were included in the multivariate model to eliminate the residual effects of the studied factors (Maldonado & Greenland, 1993). Prior to performing the multivariate linear regression, correlations among the independent variables were examined to avoid issues of multi-collinearity. Independent variables with Spearman correlation coefficients below 0.3 were considered acceptable for inclusion in the multivariate model (Supplementary Tables S1–S4). Interaction analyses, utilizing interaction terms, were conducted to investigate the modifying effect of HL on the association between fear of COVID-19 and overall KDQOL and its subscales. Models 1 and 2 involved three terms, namely X1, X2, and X1*X2, where X1 represents the main effect of fear of COVID-19, X2 represents the main effect of the modifying factor, and X1*X2 represents the interaction between the two factors.

Finally, we employed PROCESS 4.2 within SPSS to delve into the modified impact of HL on the association between fear of COVID-19 and overall KDQOL or KDCS. To enhance comprehension of the simple slope analysis, we employed figures to visually elucidate the results. These plots were created by computing the anticipated outcomes (overall KDQOL and KDCS) across three HL values: one standard deviation above the mean (+1 SD), the mean, and one standard deviation below the mean (−1 SD). All statistical analyses were performed using IBM SPSS Version 26.0 (IBM Corp., Armonk, NY, USA), with statistical significance was established at *p* < 0.05.

## Results

### Participants’ characteristics

Table 1 presents participants’ characteristics with 517 men (53.5%), 450 women (46.54%), 585 patients (60.2%) aged 18–59 years. Kidney disease quality of life was significantly varied across several variables, including age, gender, social status, medication payment ability, suspected COVID-19 symptoms, edema, hyperthyroidism, hospitalization within one month, level of physical activity, and HD vintage (*p* < 0.05). Specifically, the mental component summary (MCS) score was significantly varied by social status, medication payment ability, suspected COVID-19 symptoms, edema, hospitalization within one month, and physical activity. Similarly, the physical component summary (PCS) score was significantly varied by age, education level, marital status, social status, medication payment ability, suspected COVID-19 symptoms, hyperthyroidism, hospitalization within one month, and physical activity. Additionally, the kidney disease component summary (KDCS) score was significantly varied by age, gender, social status, medication payment ability, suspected COVID-19 symptoms, edema, hyperthyroidism, hospitalization within one-month, physical activity, and HD vintage (Table 1).

**Table 1.** Patients’ characteristics, kidney disease quality of life, and subscales (N = 972).

Variables	Total n (%)	MCS (Mean ± SD)	PCS (Mean ± SD)	KDCS (Mean ± SD)	KDQOL (Mean ± SD)
Age					
18–59	585 (60.2)	41.0 ± 9.8	34.8 ± 9.2	57.3 ± 14.9	50.8 ± 11.3
60–85	387 (39.8)	40.8 ± 10.3	32.4 ± 8.5	53.8 ± 16.4	48.1 ± 12.5
Gender					
Male	517 (53.5)	41.1 ± 9.9	34.2 ± 9.2	57.1 ± 15.8	48.7 ± 11.7
Female	450 (46.5)	40.6 ± 10.1	33.4 ± 8.9	54.5 ± 15.3	50.6 ± 11.9
Education					
Illiterate or elementary	399 (44.0)	40.7 ± 10.3	32.8 ± 8.9	56.4 ± 15.9	49.8 ± 12.3
Junior high school	281 (31.0)	41.5 ± 8.3	34.6 ± 8.2	54.9 ± 15.3	49.3 ± 11.3
Senior high school or above	226 (25.0)	41.6 ± 10.6	34.2 ± 9.4	55.1 ± 16.5	49.4 ± 12.5
Working status					
Not working	334 (34.4)	41.3 ± 7.9	33.4 ± 7.7	55.5 ± 16.8	49.4 ± 12.6
Working	638 (65.6)	40.7 ± 10.9	34.1 ± 9.6	56.2 ± 14.9	44.9 ± 11.5
Married status					
Never married	86 (8.8)	41.9 ± 9.8	36.5 ± 9.7	56.8 ± 16.2	50.9 ± 12.50
Ever married	886 (91.2)	40.8 ± 9.9	33.6 ± 8.9	55.8 ± 15.6	49.6 ± 11.8
Social status					
Low	274 (28.2)	38.3 ± 10.6	31.9 ± 9.5	49.7 ± 15.0	44.8 ± 11.5
Middle & high	698 (71.8)	42.0 ± 9.5	34.6 ± 8.7	58.4 ± 15.1	51.7 ± 11.50
Medication payment ability					
Very or fairly difficult	726 (75.9)	39.3 ± 9.4	33.2 ± 9.0	52.6 ± 14.4	47.2 ± 10.9
Very or fairly easy	231 (24.1)	46.0 ± 9.9	36.0 ± 8.6	66.0 ± 14.8	57.7 ± 11.4
S-COVID-19-S					
Without S-COVID-19-S	274 (28.2)	43.3 ± 11.1	35.7 ± 10.7	63.1 ± 15.1	55.2 ± 12.0
With S-COVID-19-S	698 (71.8)	39.8 ± 9.2	32.9 ± 7.9	52.5 ± 14.6	47.1 ± 10.9
BMI, kg/m^2^					
BMI < 24	876 (90.1)	40.8 ± 9.8	33.8 ± 9.1	56.7 ± 15.5	49.6 ± 11.8
BMI ≥ 24	96 (9.9)	41.9 ± 11.2	33.9 ± 8.1	58.1 ± 16.8	51.3 ± 12.9
Edema					
No	527 (54.2)	42.6 ± 10.7	33.5 ± 9.0	57.5 ± 16.2	51.0 ± 12.6
Yes	445 (45.8)	39.0 ± 8.6	34.2 ± 9.0	54.1 ± 14.6	48.2 ± 10.8
Hypothyroidism					
No	961 (98.9)	40.9 ± 10.0	33.8 ± 9.0	56.0 ± 15.5	49.8 ± 11.8
Yes	11 (1.1)	42.2 ± 9.1	32.6 ± 10.8	48.2 ± 19.7	44.6 ± 14.0
Hyperthyroidism					
No	930 (95.8)	40.9 ± 10.1	34.0 ± 9.1	56.3 ± 15.6	50.0 ± 11.9
Yes	41 (4.2)	42.6 ± 7.7	30.6 ± 7.3	47.5 ± 14.7	43.9 ± 11.2
Hospitalization within one month					
No	906 (93.3)	41.2 ± 9.6	34.0 ± 8.7	56.6 ± 14.9	50.3 ± 11.4
Yes	65 (6.7)	37.4 ± 13.7	31.4 ± 13.1	46.3 ± 20.6	42.4 ± 15.9
Physical activity, MET-min/wk					
Tertile 1 (MET ≤ 178)	232 (31.8)	38.0 ± 8.9	31.8 ± 8.2	52.9 ± 14.0	46.9 ± 10.2
Tertile 2 (178 < MET ≤ 912)	249 (34.2)	41.2 ± 10.8	33.2 ± 9.1	58.4 ± 14.6	51.3 ± 11.4
Tertile 3 (MET > 912)	248 (34.0)	44.5 ± 12.0	35.5 ± 9.9	62.4 ± 17.0	54.9 ± 13.1
HD vintage, years					
HD vintage ≤ 5	550 (56.7)	41.5 ± 9.8	34.4 ± 8.9	56.1 ± 15.8	50.1 ± 12.0
5 < HD vintage ≤ 10	276 (28.5)	40.5 ± 11.1	33.2 ± 9.7	57.5 ± 14.8	50.6 ± 11.6
HD vintage ≥ 10	144 (14.8)	39.6 ± 8.1	33.1 ± 8.0	52.0 ± 15.8	46.8 ± 11.6
CCI (Mean ± SD)	2.1 ± 3.0				
Fear of COVID-19 (Mean ± SD)	20.6 ± 6.0				
HL index (Mean ± SD)	25.0 ± 9.2				
DDL index (Mean ± SD)	24.1 ± 11.6				
HDK (Mean ± SD)	5.4 ± 2.5				

Abbreviation: KDQOL: kidney disease quality of life; MCS: mental component summary; PCS: physical component summary; KDCS: kidney disease component summary; S-COVID-19-S: suspected COVID-19 symptoms; BMI: body mass index; HD: hemodialysis; CCI: Charlson Comorbidity index; DDL: digital health diet literacy; HDK: hemodialysis dietary knowledge.

### Associated factors of kidney disease quality of life

Table 2 showed the results of bivariate and multivariate linear regression models of overall KDQOL. In the bivariate model, the determinants of overall KDQOLwere age, gender, social status, medication payment ability, suspected COVID-19 symptoms, BMI, edema, hypothyroidism, hyperthyroidism, hospitalization within one month, level of physical activity, HD vintage, CCI, fear of COVID-19, HL index, DDL index, and HDK. We found that medical payment ability was correlated with physical activity at *rho* = 0.31, CCI was correlated with edema at *rho* = 0.34, and HL was correlated with DDL at *rho* = 0.75 (Table S1). Therefore, edema, physical activity, and HL were analyzed in the multivariate model together with other factors that show a significant association with overall KDQOL in the bivariate model. The results show that the factor associated with higher the overall KDQOL score was social status ‘unstandardized regression coefficient’, B = 2.70 (95% confidence interval, 95% CI = 1.13–4.28, *p* = 0.001), edema B = 1.14 (95% CI = 0.01–2.88, *p* = 0.049), physical activity B = 1.33 (95% CI = 0.43–2.22, *p* = 0.004), HL B = 0.13 (95% CI = 0.06–0.21, *p* = 0.001), HDK B = 0.58 (95% CI = 0.31–0.85, *p* = 0.001). Conversely, S-COVID-19-S B = − 6.12 (95% CI = −7.66 to − 4.58, *p* = 0.001), hyperthyroidism B = −6.84 (95% CI = −10.58 to − 3.10, *p* = 0.001), hospitalization within one month B = − 6.74 (95% CI = −9.79 to −3.68, *p* = 0.001), and fear of COVID-19 B = −0.91 (95% CI = −1.03 to −0. 80, *p* = 0.001) were associated with lower the overall KDQOL score.

**Table 2.** Associated factors of kidney disease quality of life via bivariate and multivariate linear regression analysis.

Variables	KDQOL			
	Bivariate		Multivariate	
	B (95% CI)	p	B (95% CI)	p
Age				
18–59				
60–85	− 2.72 (− 4.24, − 1.20)	0.001*	− 1.26 (− 2.60, 0.09)	0.067
Gender				
Male				
Female	− 1.88 (−3.38, − 0.38)	0.014*	− 0.37 (− 170, 0.95)	0.581
Education				
Illiterate/elementary				
Junior high school	− 0.55 (− 2.39, 1.29)	0.558		
Senior high school or above	− 0.46 (− 2.43, 1.51)	0.646		
Working status				
Not working				
Working	0.48 (− 1.10, 2.05)	0.553		
Married status				
Never married				
Ever married	− 1.25 (− 3.90, 1.40)	0.354		
Social status				
Low				
Middle & high	6.82 (5.22, 8.43)	0.001*	2.70 (1.13, 4.28)	0.001*
Medication payment ability				
Very or fairly difficult				
Very or fairly easy	10.52 (8.89, 12.15)	0.001*		
S-COVID-19-S				
Without S-COVID-19-S				
With S-COVID-19-S	− 8.08 (− 9.60, − 6.56)	0.001*	− 6.12 (− 7.66, − 4.58)	0.001*
BMI, kg/m^2^				
BMI < 24				
BMI ≥ 24	1.77 (− 0.74, 4.29)	0.167	1.78 (− 0.34, 3.89)	0.100
Edema				
No				
Yes	− 2.78 (− 4.27, − 1.29)	0.001*	1.44 (0.01, 2.88)	0.049*
Hypothyroidism				
No				
Yes	− 5.20 (− 12.26, 1.87)	0.149	1.91 (− 3.99, 7.82)	0.525
Hyperthyroidism				
No				
Yes	− 6.10 (− 9.80, − 2.39)	0.001*	− 6.84 (− 10.58, − 3.10)	0.001*
Hospitalization within one month				
No				
Yes	− 7.92 (− 10.87, − 4.96)	0.001*	− 6.74 (− 9.79, − 3.68)	0.001*
Physical activity, MET-min/wk				
Tertile 1 (MET ≤ 178)				
Tertile 2 (178 < MET ≤ 912)	4.41 (2.33, 6.50)	0.001*		
Tertile 3 (MET > 912)	8.03 (5.94, 10.12)	0.001*	1.33 (0.43, 2.22)	0.004*
HD vintage, years				
HD vintage ≤ 5				
5 < HD vintage ≤ 10	0.51 (− 1.20, 2.23)	0.556		
HD vintage ≥ 10	− 3.30 (− 5.47, − 1.12)	0.003*	− 0.24 (− 1.24, 0.77)	0.644
CCI	− 0.57 (− 0.82, − 0.32)	0.001*		
Fear of COVID-19	− 1.11 (− 1.22, − 1.01)	0.001*	− 0.91 (− 1.03, − 0.80)	0.001*
HL index	0.32 (0.24, 0.40)	0.001*	0.13 (0.06, 0.21)	0.001*
DDL index	0.16 (0.09, 0.22)	0.001*		
HDK	0.72 (0.42, 1.02)	0.001*	0.58 (0.31, 0.85)	0.001*

Abbreviation: KDQOL: kidney disease quality of life; S-COVID-19-S: suspected COVID-19 symptoms; BMI: body mass index; MET: metabolic equivalent task; HD: hemodialysis; CCI: Charlson Comorbidity index; HL: health literacy; DDL: digital health diet literacy; HDK: hemodialysis dietary knowledge; B: unstandardized regression coefficient; CI: confidence interval. * The statistically significant results with a *p*-value < 0.05.

### Factors associated with subscales of kidney disease quality of life

In the bivariate analysis, the determinants of MCS were social status, medication payment ability, suspected COVID-19 symptoms, edema, hospitalization within one-month, physical activity, HD vintage, CCI, fear of COVID-19, HL index, and DDL index. We found that medical payment ability was correlated with physical activity at *rho* = 0.31, CCI was correlated with edema at *rho* = 0.34, and HL was correlated with DDL at *rho* = 0.75 (Table S2). Therefore, CCI, HL index, and physical activity were analyzed in the multivariate model together with other factors that showed a significant association with MCS in the bivariate model. The results show that factors associated with higher MCS scores were physical activity B = 1.19 (95% CI = 0.18–2.19, *p* = 0.021). Conversely, S-COVID-19 S B = −2.36 (95% CI = − 4.01 to −0.71, *p* = 0.005), hospitalization with one month B = −5.63 (95% CI = −9.04 to −2.22, *p* = 0.001), HD vintage B = −1.33 (95% CI = −2.40 to −0.26, *p* = 0.015), CCI B = − 0.43 (95% CI = −0.67 to −0.19, *p* = 0.001), and fear of COVID-19 B = −0.51 (95% CI = −0.64 to − 0.38, *p* = 0.001) were associated with lower MCS scores (Table 3).

**Table 3.** Associated factors of subscales of kidney disease quality of life via bivariate and multivariate linear regression analysis.

Variables	MCS				PCS				KDCS			
	Bivariate		Multivariate		Bivariate		Multivariate		Bivariate		Multivariate	
	B (95% CI)	p	B (95% CI)	p	B (95% CI)	p	B (95% CI)	p	B (95% CI)	p	B (95% CI)	p
Age												
18–59												
60–85	− 0.27 (−1.55, 1.02)	0.684			− 2.32 (− 3.47, −1.17)	0.001*	−1.48 (− 2.84, −1.13)	0.031*	− 3.44 (−5.44, − 1.45)	0.001*	− 2.03 (− 3.81, − 0.24)	0.026*
Gender												
Male												
Female	− 0.53 (−1.80, 0.73)	0.407			− 0.83 (− 1.98, 0.31)	0.153	− 0.83 (− 2.14, 0.47)	0.211	− 2.49 (− 4.46, − 0.52)	0.013*	− 0.33 (−2.09, 1.43)	0.715
Education												
Illiterate/elementary												
Junior high school	0.87 (− 0.62, 2.37)	0.253			1.82 (0.47, 3.17)	0.008*			− 1.48 (− 3.91, 0.94)	0.230		
Senior high school or above	0.98 (0.62, 2.57)	0.231			1.45 (0.01, 2.90)	0.048*	− 0.84 (−1.66, − 0.01)	0.047*	− 1.30 (− 3.89, 1.29)	0.325		
Working status												
Not working												
Working	− 0.64 (−1.97, 0.68)	0.339			0.70 (− 0.49, 1.90)	0.249			0.71 (− 1.26, 2.78)	0.503		
Married status												
Never married												
Ever married	−1.09 (− 3.30, 1.13)	0.335			− 2.94 (− 4.94, − 0.95)	0.004*	− 0.98 (− 3.28, 1.32)	0.403	− 0.91 (− 4.39, 2.56)	0.606		
Social status												
Low												
Middle & high	3.62 (2.24, 5.00)	0.001*	1.06 (− 0.66, 2.77)	0.226	2.69 (1.44, 3.95)	0.001*	0.96 (− 0.67, 2.60)	0.247	8.66 (6.55, 10.78)	0.001*	3.46 (1.37, 5.55)	0.001*
Medication payment ability												
Very or fairly difficult												
Very or fairly easy	6.67 (5.26, 8.07)	0.001*			2.73 (1.41, 4.05)	0.001*			13.42 (11.36, 15.57)	0.001*		
S-COVID-19-S												
Without S-COVID-19-S												
With S-COVID-19-S	− 3.53 (−4.86, −2.20)	0.001*	− 2.36 (− 4.01, − 0.71)	0.005*	− 2.74 (− 3.95, − 1.53)	0.001*	− 0.77 (− 2.23, 0.68)	0.298	− 10.55 (− 12.54, − 8.55)	0.001*	− 8.21 (−10.25, − 6.17)	0.001*
BMI, kg/m^2^												
BMI < 24												
BMI ≥ 24	1.14 (−0.96, 3.25)	0.287			0.09 (−1.82, 1.99)	0.925			2.40 (− 0.91, 5.70)	0.155	2.14 (− 0.67, 4.95)	0.135
Edema												
No												
Yes	−3.63 (− 4.87, −2.39)	0.001*			0.68 (− 0.47, 1.82)	0.245			− 3.44 (− 5.40, −1.48)	0.001*	2.46 (0.56, 4.37)	0.011*
Hypothyroidism												
No												
Yes	1.24 (− 470, 7.18)	0.682			−1.20 (− 6.58, 4.17)	0.661			− 7.81 (− 18.08, 1.47)	0.099	1.06 (− 6.77, 8.90)	0.790
Hyperthyroidism												
No												
Yes	1.78 (−1.35, 4.91)	0.264			− 3.39 (− 6.21, − 0.57)	0.019*	− 5.07 (−8.57, − 1.60)	0.005*	− 8.75 (− 13.61, − 3.89)	0.001*	− 9.94 (− 14.91, − 4.98)	0.001*
Hospitalization within one month												
No												
Yes	−3.78 (−6.28, −1.27)	0.003*	− 5.63 (− 9.04, −2.22)	0.001*	− 2.62 (− 4.89, − 0.35)	0.024*	− 4.77 (− 7.98, − 1.55)	0.004*	− 10.28 (− 14.16, − 6.40)	0.001*	− 6.93 (− 10.98, − 2.87)	0.001*
Physical activity, MET-min/wk												
Tertile 1 (MET ≤ 178)												
Tertile 2 (178 < MET ≤ 912)	3.13 (1.22, 5.05)	0.001*			1.42 (− 0.21, 3.05)	0.087			5.48 (2.74, 8.22)	0.001*		
Tertile 3 (MET > 912)	6.50 (4.59, 8.42)	0.001*	1.19 (0.18, 2.19)	0.021*	3.70 (2.07, 5.33)	0.001*	1.44 (0.56, 2.32)	0.001*	9.51 (6.77, 12.26)	0.001*	1.41 (0.23, 2.60)	0.020*
HD vintage, years												
HD vintage ≤ 5												
5 < HD vintage ≤ 10	−1.08 (−2.52, 0.37)	0.143			− 1.20 (− 2.50, 0.11)	0.072			1.36 (− 0.89, 3.61)	0.237		
HD vintage ≥ 10	−1.96 (−3.79, −0.12)	0.036*	−1.33 (− 2.40, − 0.26)	0.015*	− 1.25 (− 2.90, 0.41)	0.141	− 0.92 (− 1.90, 0.06)	0.066	− 4.15 (−7.00, − 1.29)	0.004*	0.33 (− 1.00, 1.66)	0.626
CCI	− 0.69 (− 0.90, − 0.49)	0.001*	− 0.43 (−0.67, − 0.19)	0.001*	− 0.22 (− 0.41, − 0.03)	0.021*	0.025 (−0.24, 0.29)	0.856	− 0.63 (− 0.95, − 0.30)	0.001*		
Fear of COVID-19	− 0.54 (− 0.64, − 0.44)	0.001*	− 0.51 (− 0.64, − 0.38)	0.001*	− 0.33 (− 0.42, − 0.23)	0.001*	− 0.22 (− 0.33, − 0.10)	0.001*	− 1.45 (− 1.59, − 1.31)	0.001*	− 1.19 (− 1.34, − 1.03)	0 .001*
HL index	0.12 (0.05, 0.19)	0.001*	0.06 (− 0.02, 0.14)	0.122	0.18 (0.12, 0.24)	0.001*	0.13 (0.05, 0.20)	0.001*	0.40 (0.30, 0.51)	0.001*	0.15 (0.05, 0.25)	0.003*
DDL index	0.07 (0.01, 0.12)	0.017*			0.09 (0.04, 0.14)	0.001*			0.19 (0.11, 0.28)	0.001*		
HDK	0.15 (− 0.10, 0.40)	0.242			0.09 (− 0.14, 0.31)	0.461			1.02 (0.63, 1.40)	0.001*	0.80 (0.44, 1.16)	0 .001*

Abbreviation: MCS: mental component summary; PCS: physical component summary; KDCS: kidney disease component summary; S-COVID-19-S: suspected COVID-19 symptoms; BMI: body mass index; MET: metabolic equivalent task; HD: hemodialysis; CCI: Charlson Comorbidity index; HL: health literacy; DDL: digital health diet literacy; HDK: hemodialysis dietary knowledge; B: unstandardized regression coefficient; CI: confidence interval.

* The statistically significant results with a *p*-value < 0.05.

In the bivariate analysis, the determinants of PCS were age, gender, education, married status, social status, medication payment ability, S-COVID-19 S, hyperthyroidism, hospitalization within one-month, physical activity, HD vintage, CCI, fear of COVID-19, HL index, and DDL index. We found that medical payment ability was correlated with physical activity at *rho* = 0.31, and HL was correlated with DDL at *rho* = 0.75 (Table S3). Therefore, CCI and HL were analyzed in the multivariate model together with other factors that showed a significant association with PCS in the bivariate model. The results show that factors associated with higher PCS scores were physical activity B = 1.44 (95% CI = 0.56–2.32, *p* = 0.001) and HL B = 0.13 (95% CI = 0.05–0.20, *p* = 0.001). Conversely, age B = −1.48 (95%CI = −2.84 to −1.13, *p* = 0.031), education B = −0.84 (95% CI = −1.66 to −0.01, *p* = 0.047), hyperthyroidism B = −5.07 (95% CI = −9.57 to −1.60, *p* = 0.005), hospitalization within one month B = −4.77 (95% CI = −7.98 to −1.55, *p* = 0.004), and fear of COVID-19 B = −0.22 (95% CI = −0.33 to −0.10, *p* = 0.001) were associated with lower PCS scores. However, we didn’t find a significant association between HL and either MCS scores or PCS scores (Table 3).

In the bivariate analysis, the determinants of KDCS were age, gender, social status, medication payment ability, S-COVID-19-S, BMI, edema, hypothyroidism, hyperthyroidism, hospital within one-month, physical activity, HD vintage, CCI, fear of COVID-19, HL index, DDL index, and HDK. We found that medical payment ability was correlated with physical activity at *rho* = 0.31, CCI was correlated with edema at *rho* = 0.34, and HL was correlated with DDL at *rho* = 0.75 (Table S4). Therefore, edema, physical activity, and HL were analyzed in the multivariate model together with other factors that show a significant association with QOL in the bivariate model. The results show that factors associated with higher KDCS scores were social status B = 3.46 (95%CI = 1.37–5.55, *p* = 0.001), edema B = 2.46 (95% CI = 0.56–4.37, *p* = 0.011), physical activity B = 1.41 (95% CI = 0.23–2.60, *p* = 0.02), HL B = 0.15 (95% CI = 0.05–0.15, *p* = 0.003), and HDK B = 0.80 (95%CI = 0.44–1.16, *p* = 0.001). Conversely, age B = −2.03 (95%CI = −3.81 to −0.24, *p* = 0.026), S-COVID-19 S B = −8.21 (95% CI = −10.25 to −6.17, *p* = 0.001), hyperthyroidism B = −9.94 (95%CI = −14.91 to −4.98, *p* = 0.001), hospitalization within one month B = −6.93 (95% CI = −10.98 to −2.97, *p* = 0.001), and fear of COVID-19 B = −1.19 (95% CI = −1.34 to −1.03, *p* = 0.001) were correlated with lower scores of KDCS (Table 3).

### Interaction of health literacy with fear of COVID-19 on kidney disease quality of life

Table 4 illustrates how the association between fear of COVID-19 and KDQOL was modified by HL. Specifically, patients with one score increment in both fear of COVID-19 and health literacy exhibited higher overall KDQOL B = 0.011 (95%CI = 0.000–0.021, *p* = 0.042), and KDCS B = 0.015 (95%CI  = 0.001–0.028, *p* = 0.038).

**Table 4.** Interaction of health literacy with fear of COVID-19 on KDQOL and KDCS.

	KDQOL*		KDCS *	
	B (95% CI)	*p*	B (95% CI)	*p*
Fear of COVID-19 (lowest score) x HL (lowest score)	ref		ref	
Fear of COVID-19 (one score increment) x HL (lowest score)	− 1.182 (− 1.466, −0.898)	0.001	− 1.550 (− 1.927, − 1.174)	0.001
Fear of COVID-19 (lowest score) x HL (one score increment)	− 0.081 (− 0.302, 0.139)	0.469	− 0.140 (− 0.433, 0.152)	0.347
Fear of COVID-19 (one score increment) x HL (one score increment)	0.011 (0.000, 0.021)	0.042	0.015 (0.001, 0.028)	0.038

Abbreviation: KDQOL: kidney disease quality of life, KDCS: kidney disease component summary; HL: health literacy; CI: confidence interval. * These models were adjusted for age, gender, social, S-COVID-19 S, hyperthyroidism, Physical activity, HD vintage, edema, HDK, hospitalization, BMI, and hypothyroidism.

**Figure 1.**
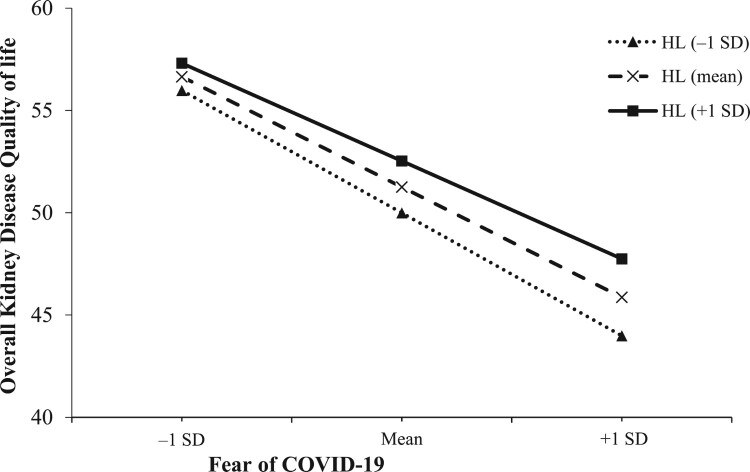
Simple slope plot of interaction between health literacy and fear of COVID-19 on overall kidney disease quality of life. SD, standard deviation; HL, health literacy.

Figure 1 illustrates the results of the simple slope analysis, revealing that as HL increased, the association between fear of COVID-19 and overall KDQOL diminished. A higher HL score mitigates the negative impact of COVID-19-related fear on overall KDQOL, transitioning from B = −1.01 (95%CI = −1.16 to −0.86, *p* = 0.001) at one standard deviation below the mean, to B = −0.91 (95%CI = −1.03 to −0.79, *p* = 0.001) at the mean, and B = −0.81 (95%CI = −0.96 to −0.65, *p* = 0.001) at one standard deviation above the mean.

**Figure 2.**
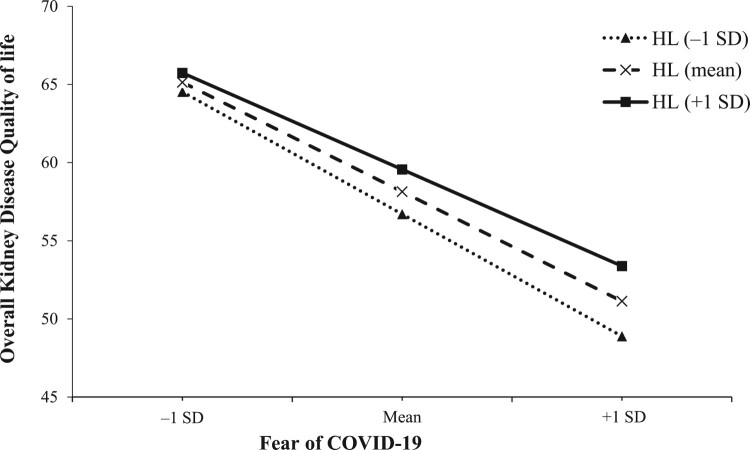
Simple slope plot of interaction between health literacy and fear of COVID-19 on kidney disease component summary. SD, standard deviation; HL, health literacy.

Similarly, Figure 2 demonstrates that as HL increased, the association between fear of COVID-19 and KDCS diminished. A higher HL score mitigates the negative impact of COVID-19-related fear on KDCS, transitioning from B = −1.32 (95%CI = −1.52 to −1.12, *p* = 0.001) at one standard deviation below the mean, to B = −1.18 (95%CI = −1.34 to −1.03, *p* = 0.001) at the mean, and B = −1.04 (95%CI = −1.25 to −0.84, *p* = 0.001) at one standard deviation above the mean.

## Discussion

In the current study, the results showed that more fear of COVID-19 was associated with a lower overall KDQOL score and its subscales (PCS, MCS, and KDCS), indicating the detrimental impact of fear on the well-being of these patients. This finding is consistent with previous studies that have examined the impact of fear of COVID-19 on the quality of life in hemodialysis patients (Parlak & Akgün Şahin, 2021), as well as in other populations, such as inpatients, outpatients, general publics, and healthcare workers (Abdelghani et al., 2022; Baysal et al., 2022; Demirbas & Kutlu, 2022; Nguyen et al., 2021).

Fear of COVID-19 can affect the quality of life of hemodialysis patients through increased stress, anxiety, and psychological distress (Beaini et al., 2021; Lee et al., 2020). The fear and anxiety surrounding the pandemic can lead to stress and emotional strain, which can negatively impact their mental health and overall quality of life. Hemodialysis patients were particularly vulnerable when they accessed the treatment weekly (2-3 times a week) in the dialysis center which may cause more fear, panic, and increased risk of infection (Lee et al., 2020). During the pandemic, patients with the less social support, limited access to resources, and feelings of loneliness and isolation, all of which can contribute to a decline in their quality of life (Hwang et al., 2020). Furthermore, Fear of COVID-19 further exacerbates existing health concerns among hemodialysis patients (Nadort et al., 2022). The stress and anxiety associated with the pandemic can impact patient’s physical well-being, potentially leading to disrupted sleep patterns, increased fatigue, and decreased overall functional capacity (Lee et al., 2020). This further diminishes their quality of life and negatively impacts their ability to cope with the challenges of managing their kidney disease. By identifying fear of COVID-19, and its impact on quality of life, our study emphasizes the need for targeted interventions and support strategies to mitigate the negative impact of COVID-19-related fear in hemodialysis patients.

The higher HL levels were associated with higher overall KDQOL scores and all subscales. HL further diminishes the negative impact of COVID-19-related fear on overall KDQOL and KDCS components. HL was found to play a critical role in shaping health outcomes and patients’ ability to effectively manage their health conditions (Chow et al., 2024; Liu et al., 2020).

During the data analysis, the protective effect of HL against the negative impact of fear on the MCS and PCS of quality of life was not found in the current study. This may be influenced by several factors. In contrast, the MCS and PCS components of the KDQOL™−36 are more generic measures of mental and physical well-being and may be influenced by a wider range of factors beyond fear and HL alone. Take into account, HL may have a more specific influence on certain dimensions of QOL, particularly those related to kidney disease and its management. In light of a previous study, HL was proven to reduce anxiety among hemodialysis patients (Yoon & Lee, 2023). This finding suggests that interventions aimed at improving HL in hemodialysis patients may have a positive impact on their quality of life, especially in the face of fear and anxiety related to COVID-19.

Besides, we found that S-COVID-19-S was negatively associated with overall KDQOL and all subscales. This result is similar to the previous study in the outpatients (Nguyen, Nguyen et al., 2020). Suspected COVID-19 symptoms, such as fever, cough, fatigue, dyspnea, myalgia, and others, can cause physical discomfort and hinder daily activities (Sharp et al., 2021). These symptoms can directly impact a person's ability to engage in routine tasks. Experiencing symptoms associated with COVID-19 can induce anxiety, fear, and psychological distress. The fear of having a potentially severe illness or the uncertainty surrounding the symptoms can negatively affect a person's mental well-being. Taken together, these lead to a decrease in their quality of life.

Among the clinical factors considered, edema had a negative impact on the overall KDQOL and its subscales in hemodialysis patients, apart from the PCS subscale. It has been observed that individuals dealing with chronic edema experience diminished health conditions and a more pronounced influence on various aspects of HRQOL (Moffatt et al., 2017). Our findings are consistent with a previous study (Pretto et al., 2020) conducted on hemodialysis patients, where a connection between edema and a diminished quality of life was established. The negative effect of edema on quality of life suggests that the presence of edema is associated with poorer well-being and functioning in various aspects of the patients’ lives. However, it is important to note that the impact of edema on quality of life may vary across different subscales. In the case of the PCS subscale, which primarily focuses on the physical health and functioning of individuals, the presence of edema may not show a significantly negatively association. This could imply that the impact of edema may show the impact on other aspects of quality of life, such as mental well-being, emotional functioning, and the burden of kidney disease-related symptoms.

Moreover, we also explored that a longer HD vintage and a higher CCI score were associated with a lower overall KDQOL score. Specifically, patients with more than 10 years of HD vintage had lower quality of life across all subscales, except PCS. A previous study showed a significant decrease in the PCS score as dialysis vintage lengthened, while no association was found with a change in the MCS score. The MCS score declined over time in older patients, particularly those aged 80 years and older, after 2 years of follow-up (Ishiwatari et al., 2020). However, in our population, individuals aged 18–59 accounted for 60.2%. Therefore, HD vintage seems to have a greater effect on MCS than PCS in young HD patients. Conversely, intensive physical activity was positively associated with overall KDQOL. Physical activity has also been proven to relate to a higher overall KDQOL in other populations (Anokye et al., 2012; Puciato et al., 2018; Wei et al., 2022), specifically in hemodialysis patients with comorbidities, where improvement can be achieved through early intervention involving regular moderate-intensity physical activity (Wu et al., 2022). Recent studies have also shown the positive effects of intradialytic exercise on quality of life (Greenwood et al., 2021). Therefore, we need to encourage HD patients to engage in more physical activity.

The results of this study have important implications for healthcare professionals, policymakers, and patient advocacy groups in designing tailored interventions and educational programs to address the unique needs of hemodialysis patients during the COVID-19 pandemic and beyond. Recognizing the significant impact of COVID-19-related fear on overall KDQOL, healthcare providers can prioritize interventions. These may include providing protective measures for both COVID-19 and non-COVID-19 patients, implementing vaccination programs for all patients, and offering support to alleviate fear and anxiety within this population (Chirico & Teixeira da Silva, 2023). Furthermore, considering the potential moderating role of HL, interventions aimed at its enhancement may positively influence quality of life. To achieve this, patient education programs tailored specifically to hemodialysis patients should be implemented. These programs ought to encompass crucial topics, including kidney disease, hemodialysis treatment procedures, dietary restrictions, medication management, and recognizing signs of complications. Additionally, one-on-one counseling sessions should be offered by healthcare providers, nurses, dietitians, and pharmacists to address patients’ specific questions and concerns regarding their treatment plans, medications, and lifestyle adjustments (Murdeshwar & Anjum, 2024). Peer support groups can also be facilitated, allowing hemodialysis patients to connect with others facing similar challenges, exchange experiences, and offer practical advice for managing their condition. Regular assessments of patients’ health literacy levels should be conducted to pinpoint areas for improvement, enabling tailored educational interventions (Dineen-Griffin et al., 2019).

Despite its contributions, this study has some limitations. First, the cross-sectional design of the study limits the ability to establish causal relationships between the variables of interest. Longitudinal studies are suggested that will provide more robust evidence on the temporal associations between fear of COVID-19, HL, and KDQOL in hemodialysis patients. Secondly, the study was conducted in Vietnam, which may limit the generalizability of the findings to other cultural and healthcare contexts. Finally, the study relied on self-reported measures, which are subject to recall and social desirability biases. The inclusion of objective measures and additional sources of data would enhance the validity of the findings. Future research should aim to replicate these findings in diverse populations and settings, utilizing more comprehensive and disease-specific questionnaires for HL.

## Conclusions

In this study, fear of COVID-19, symptoms like COVID-19, hemodialysis vintage, and comorbid conditions were negatively associated with KDQOL and subscales. Fortunately, HL, DDL, and physical activity were positively associated with overall KDQOL and its subscales. In addition, a higher health literacy score was linked to a mitigation of the negative impact fear of COVID-19 on both overall KDQOL and KDCS. These results provide significant evidence supporting the implementation of targeted measures aimed at alleviating fear and improve quality of life in hemodialysis patients.

## Institutional Review Board Statement

The study was conducted in accordance with the Declaration of Helsinki and was approved by an Institutional Review Board/Ethics committee. See details under Methods

## Supplementary Material

Supplementary.docx
