# Supplementary material for: Associations of COVID-19-related fear with kidney disease quality of life and its subscales among hemodialysis patients as modified by health literacy: a multi-hospital survey
Source: Health Psychol Behav Med. 2024 Jul 12;12(1):2376585. doi: 10.1080/21642850.2024.2376585 (PMC11249155; doi:10.1080/21642850.2024.2376585)
Supplement: Supplementary.docx [file RHPB_A_2376585_SM6684.docx]

Table S1: The correlations between the independent variable ( KDQOL)

|  | Age | Gender | Social | Payment | S-COVID-19-S | Hyper  thyroidism | Physical acitivy | HD vintage | CCI | Fear of COVID 19 | HL | DDL | HDK | Hospita  lization | BMI | Edema | Hypo  thyroidism |
| --- | --- | --- | --- | --- | --- | --- | --- | --- | --- | --- | --- | --- | --- | --- | --- | --- | --- |
| Age | 1 |  |  |  |  |  |  |  |  |  |  |  |  |  |  |  |  |
| Gender | 0.052 | 1 |  |  |  |  |  |  |  |  |  |  |  |  |  |  |  |
| Social | −0.032 | −0.044 | 1 |  |  |  |  |  |  |  |  |  |  |  |  |  |  |
| Payment | 0.043 | −0.047 | 0.285 | 1 |  |  |  |  |  |  |  |  |  |  |  |  |  |
| S-COVID-19-S | 0.089 | 0.099 | −0.121 | −0.155 | 1 |  |  |  |  |  |  |  |  |  |  |  |  |
| Hyperthyroidism | −0.077 | 0.071 | −0.062 | 0.014 | 0.045 | 1 |  |  |  |  |  |  |  |  |  |  |  |
| Physical Activity | −0.047 | −0.075 | 0.249 | 0.3116 | −0.221 | −0.015 | 1 |  |  |  |  |  |  |  |  |  |  |
| HD vintage | 0.001 | 0.056 | 0.005 | −0.087 | −0.052 | 0.143 | −0.055 | 1 |  |  |  |  |  |  |  |  |  |
| CCI | 0.188 | 0.046 | −0.143 | −0.221 | −0.263 | 0.019 | −0.400 | −0.009 | 1 |  |  |  |  |  |  |  |  |
| Fear of COVID-19 | 0.015 | 0.058 | −0.140 | −0.255 | 0.109 | 0.066 | −0.173 | 0.056 | 0.155 | 1 |  |  |  |  |  |  |  |
| HL | −0.164 | −0.036 | 0.143 | 0.206 | −0.016 | 0.036 | 0.199 | −0.034 | −0.126 | −0.123 | 1 |  |  |  |  |  |  |
| DDL | −0.149 | −0.071 | 0.067 | 0.162 | −0.013 | 0.049 | 0.178 | −0.021 | −0.180 | −0.051 | 0.750 | 1 |  |  |  |  |  |
| HDK | −0.029 | −0.048 | 0.087 | 0.131 | −0.058 | 0.077 | 0.084 | 0.139 | −0.099 | −0.079 | 0.233 | 0.212 | 1 |  |  |  |  |
| Hospitalization | −0.008 | 0.007 | −0.089 | −0.085 | −0.055 | 0.026 | 0.010 | 0.069 | 0.110 | 0.107 | −0.036 | 0.014 | −0.009 | 1 |  |  |  |
| BMI | 0.005 | −0.029 | 0.000 | 0.055 | −0.017 | −0.035 | 0.051 | 0.039 | 0.024 | −0.042 | −0.059 | −0.046 | −0.038 | 0.037 | 1 |  |  |
| Edema | 0.020 | 0.053 | −0.168 | −0.217 | 0.192 | −0.060 | −0.326 | 0.032 | 0.341 | 0.025 | −0.096 | −0.138 | −0.165 | 0.126 | 0.028 | 1 |  |
| Hypothyroidism | −0.047 | −0.002 | −0.084 | −0.015 | 0.053 | 0.074 | −0.049 | −0.001 | 0.059 | 0.049 | 0.022 | 0.023 | 0.027 | −0.029 | −0.035 | −0.020 | 1 |

Abbreviation: KDQOL, kidney disease quality of life; S-COVID-19-S, suspected COVID-19 symptoms; HD, Hemodialysis; CCI, Charlson Comorbidity Index; HL, Health literacy; DDL, digital health diet literacy; HDK, hemodialysis dietary knowledge; BMI, body mass index.

Table S2: The correlations between the independent variable ( MCS)

|  | Socail | Medication | S-COVID-19 | Edema | Hospital | PA | Hdvintage | CCI | Fear | HL | DDL |
| --- | --- | --- | --- | --- | --- | --- | --- | --- | --- | --- | --- |
| Socail | 1 |  |  |  |  |  |  |  |  |  |  |
| Medication | 0.285 | 1 |  |  |  |  |  |  |  |  |  |
| S-COVID-19 | − 0.121 | − 0.155 | 1 |  |  |  |  |  |  |  |  |
| Edema | − 0.168 | − 0.217 | 0.192 | 1 |  |  |  |  |  |  |  |
| Hospital | − 0.089 | − 0.085 | − 0.055 | 0.126 | 1 |  |  |  |  |  |  |
| PA | 0.249 | 0.316 | − 0.221 | − 0.326 | 0.01 | 1 |  |  |  |  |  |
| Hdvintage | 0.005 | − 0.087 | − 0052 | 0.032 | 0.069 | − 0.055 | 1 |  |  |  |  |
| CCI | − 0.143 | − 0.221 | 0.263 | 0.341 | 0.11 | − 0.4 | − 0.009 | 1 |  |  |  |
| Fear | − 0.14 | − 0.255 | 0.109 | 0.025 | 0.107 | − 0.173 | 0.056 | 0.155 | 1 |  |  |
| HL | 0.143 | 0.206 | − 0.016 | − 0.096 | − 0.036 | 0.199 | − 0.034 | − 0.126 | − 0.123 | 1 |  |
| DDL | 0.067 | 0.162 | − 0.013 | − 0.138 | 0.14 | 0.178 | − 0.021 | − 0.18 | − 0.051 | 0.75 | 1 |

Abbreviation:MCS, mental component summary; S-COVID-19-S, suspected COVID-19 symptoms; HD, Hemodialysis; CCI, Charlson Comorbidity Index; HL, Health literacy; DDL, digital health diet literacy.

Table S3: The correlations between the independent variable (PCS)

|  | Age | Gender | Education | Married status | Social status | Medication status | S-COVID-19-S | Hyperthyroidism | Physical | HD vintage | CCI | Fear of Covid | HL | DDL | Hospitalization |
| --- | --- | --- | --- | --- | --- | --- | --- | --- | --- | --- | --- | --- | --- | --- | --- |
| Age | 1 |  |  |  |  |  |  |  |  |  |  |  |  |  |  |
| Gender | 0.052 | 1 |  |  |  |  |  |  |  |  |  |  |  |  |  |
| Education | −0.106 | −0.089 | 1 |  |  |  |  |  |  |  |  |  |  |  |  |
| Married status | 0.194 | 0.007 | −0.092 | 1 |  |  |  |  |  |  |  |  |  |  |  |
| Social status | −0.032 | −0.044 | 0.335 | 0.022 | 1 |  |  |  |  |  |  |  |  |  |  |
| Medication payment | 0.043 | −0.047 | 0.199 | 0.024 | 0.285 | 1 |  |  |  |  |  |  |  |  |  |
| S-COVID-19-S | 0.089 | 0.099 | 0.031 | 0.043 | −0.121 | −0.155 | 1 |  |  |  |  |  |  |  |  |
| Hyperthyroidisms | −0.077 | 0.071 | 0.024 | −0.007 | −0.062 | 0.014 | 0.045 | 1 |  |  |  |  |  |  |  |
| Physical activity | −0.047 | −0.075 | 0.195 | −0.017 | 0.249 | 0.316 | −0.221 | −0.015 | 1 |  |  |  |  |  |  |
| HD vintage | 0.001 | 0.056 | −0.005 | 0.028 | 0.005 | −0.087 | −0.052 | 0.143 | −0.055 | 1 |  |  |  |  |  |
| CCI | 0.188 | 0.046 | −0.092 | 0.127 | −0.143 | −0.221 | 0.263 | 0.019 | −0.400 | −0.009 | 1 |  |  |  |  |
| Fear of COVID19 | 0.015 | 0.058 | −0.024 | 0.039 | −0.140 | −0.255 | 0.109 | 0.066 | −0.173 | 0.056 | 0.155 | 1 |  |  |  |
| Hl | −0.164 | −0.036 | 0.235 | −0.047 | 0.143 | 0.206 | −0.016 | 0.036 | 0.199 | −0.034 | −0.126 | −0.123 | 1 |  |  |
| DDL | −0.149 | −0.071 | 0.255 | −0.069 | 0.067 | 0.162 | −0.013 | 0.049 | 0.178 | −0.021 | −0.180 | −0.051 | 0.750 | 1 |  |
| Hospitalization | −0.008 | 0.007 | −0.008 | 0.025 | −0.089 | −0.085 | −0.055 | 0.026 | 0.010 | 0.069 | 0.110 | 0.107 | −0.036 | 0.014 | 1 |

Abbreviation:PCS, physical component summary; S-COVID-19-S, suspected COVID-19 symptoms; HD, Hemodialysis; CCI, Charlson Comorbidity Index; HL, Health literacy; DDL, digital health diet literacy.

Table S4: The correlations between the independent variable (KDCS)

|  | Age | Gender | Social | Payment | S-COVID-19-S | Hyper  thyroidism | Physical acitivy | HD vintage | CCI | Fear of COVID 19 | HL | DDL | HDK | Hospita  lization | BMI | Edema | Hypo  thyroidism |
| --- | --- | --- | --- | --- | --- | --- | --- | --- | --- | --- | --- | --- | --- | --- | --- | --- | --- |
| Age | 1 |  |  |  |  |  |  |  |  |  |  |  |  |  |  |  |  |
| Gender | 0.052 | 1 |  |  |  |  |  |  |  |  |  |  |  |  |  |  |  |
| Social | −0.032 | −0.044 | 1 |  |  |  |  |  |  |  |  |  |  |  |  |  |  |
| Payment | 0.043 | −0.047 | 0.285 | 1 |  |  |  |  |  |  |  |  |  |  |  |  |  |
| S-COVID-19-S | 0.089 | 0.099 | −0.121 | −0.155 | 1 |  |  |  |  |  |  |  |  |  |  |  |  |
| Hyperthyroidism | −0.077 | 0.071 | −0.062 | 0.014 | 0.045 | 1 |  |  |  |  |  |  |  |  |  |  |  |
| Physical Activity | −0.047 | −0.075 | 0.249 | 0.3116 | −0.221 | −0.015 | 1 |  |  |  |  |  |  |  |  |  |  |
| HD vintage | 0.001 | 0.056 | 0.005 | −0.087 | −0.052 | 0.143 | −0.055 | 1 |  |  |  |  |  |  |  |  |  |
| CCI | 0.188 | 0.046 | −0.143 | −0.221 | −0.263 | 0.019 | −0.400 | −0.009 | 1 |  |  |  |  |  |  |  |  |
| Fear of COVID-19 | 0.015 | 0.058 | −0.140 | −0.255 | 0.109 | 0.066 | −0.173 | 0.056 | 0.155 | 1 |  |  |  |  |  |  |  |
| HL | −0.164 | −0.036 | 0.143 | 0.206 | −0.016 | 0.036 | 0.199 | −0.034 | −0.126 | −0.123 | 1 |  |  |  |  |  |  |
| DDL | −0.149 | −0.071 | 0.067 | 0.162 | −0.013 | 0.049 | 0.178 | −0.021 | −0.180 | −0.051 | 0.750 | 1 |  |  |  |  |  |
| HDK | −0.029 | −0.048 | 0.087 | 0.131 | −0.058 | 0.077 | 0.084 | 0.139 | −0.099 | −0.079 | 0.233 | 0.212 | 1 |  |  |  |  |
| Hospitalization | −0.008 | 0.007 | −0.089 | −0.085 | −0.055 | 0.026 | 0.010 | 0.069 | 0.110 | 0.107 | −0.036 | 0.014 | −0.009 | 1 |  |  |  |
| BMI | 0.005 | −0.029 | 0.000 | 0.055 | −0.017 | −0.035 | 0.051 | 0.039 | 0.024 | −0.042 | −0.059 | −0.046 | −0.038 | 0.037 | 1 |  |  |
| Edema | 0.020 | 0.053 | −0.168 | −0.217 | 0.192 | −0.060 | −0.326 | 0.032 | 0.341 | 0.025 | −0.096 | −0.138 | −0.165 | 0.126 | 0.028 | 1 |  |
| Hypothyroidism | −0.047 | −0.002 | −0.084 | −0.015 | 0.053 | 0.074 | −0.049 | −0.001 | 0.059 | 0.049 | 0.022 | 0.023 | 0.027 | −0.029 | −0.035 | −0.020 | 1 |

Abbreviation: KDCS, kidney disease component summary; S-COVID-19-S, suspected COVID-19 symptoms; HD, Hemodialysis; CCI, Charlson Comorbidity Index; HL, Health literacy; DDL, digital health diet literacy; HDK, hemodialysis dietary knowledge; BMI, body mass index.
